# American Association of Obstetricians and Gynecologists

**Published:** 1897-05

**Authors:** 


### Society Proceedings.

### AMERICAN ASSOCIATION OF OBSTETRICIANS AND GYNECOLOGISTS.

### ABDOMINAL SECTION VS. THE VAGINAL ROUTE IN THE TREATMENT OF PERI-UTERINE SEPTIC DISEASES.[1]

DR. L. S. McMURTRY, of Louisville: Mr. President—The subject which the papers of Drs. Davis, Dunning and Fish have dealt with is one the importance of which cannot be overrated. I have strong convictions on the subject, and, at the same time, there are men whose judgment and surgical ability we cannot question from whom I differ as to methods of procedure. The difference is very great and vital, and it becomes necessary for us to take the whole situation carefully in mind and discuss it fairly, thoroughly and impartially. As I understand it, the question, briefly stated, that presents itself to us is this—namely, whether the best method of treating inflammatory diseases of the pelvic organs can be obtained by a suprapubic abdominal section or by the vaginal route.

In the first place, the surgical approach to the pelvic organs by the vagina is not new. You all remember that Battey's operations at first were done through the vault of the vagina. These opera-

1. Discussion on the papers of Drs. Davis, Dunning, and Fish, at Richmond, Va., September 22, 1896. From Transactions, Vol. IX., p. 174 *et seq.*

tions were what he called normal ovariotomies, and all these years that pelvic surgery has been practised so successfully pelvic surgeons have recognised, in certain cases in which an immediate life-saving procedure was necessary and a radical operation could not be borne, that vaginal incision and drainage was a valuable surgical procedure; hence there is nothing really new, *per se*, about the question of vaginal operations. The only question that is new in that relation is as to the removal of the uterus. The operations that have been done for suppurative pelvic disease by abdominal section have been difficult and trying. Everyone engaged in this work has recognised that the surgical treatment of suppurative inflammatory disease of the adnexæ is among the most difficult of all surgical operations. Any method that will simplify or improve the procedure, rendering it easier of accomplishment and more satisfactory in its results, will be welcome.

It is interesting here to notice the origin of the operation and the manner in which it has presented itself to us in this country. French surgery was for a long time ascendant in the world, particularly in the days of Velpeau, Nélaton, and other men who were leaders in surgery. Abdominal section never flourished in France, and Parisians are not successful operators above the pubes. They have always, in their gynecic surgery, been working in the cervix and through the vagina, and while we were doing in this country and in England abdominal section for suppurative pelvic disease, it was not a success in France. Péan, Segond, Richelot, Doyen and Pozzi have come to the front and have advocated the vaginal method of extirpation of the uterus in order to drain the pelvis and cure suppurative pelvic disease. This method extended to Brussels, which is closely allied to Paris, where the same language is spoken and the profession is as largely controlled in its surgical opinion by Paris as though it was only across the Seine. Dr. Jacobs, of Brussels, an expert operator, became a convert to the idea of Segond, Péan and Richelot, and began to do this operation. Dr. Henrotin, of Chicago, who is a native of Belgium, became an exponent of the operation in this country. Dr. Jacobs was introduced in this country by Dr. Henrotin. His visit was followed a year later by that of Segond.

The introduction of this method was cordially received, as might have been expected, by that class of surgeons in America who are always looking out for novelties in surgical operations and are extreme exponents of the restless spirit of surgery. And it is being made to appear that men who resort to abdominal section for inflammatory disease of the pelvic organs, who remove diseased structures and nothing else, are behind the time. This recital of recent history has a bearing upon the discussion of this subject which cannot be ignored. The personal equation in these operations is what determines the choice of methods. You know we have had in America, all these years, men who never operated much suprapubically, but were always ready to operate on the cervix, to curette the uterus and work in that direction. The introduction of this operation furnished to that particular school of American surgeons a field most welcome and attractive. They extirpated the uterus. If a man has been accustomed to operate through the vagina, his entire surgical training has prepared him for vaginal hysterectomy. If he has learned to operate successfully suprapubically, I do not think he will ever abandon it to take up the other operative procedure. In my judgment a surgical operation should be based upon and directed along those lines which will secure the recovery of the patient with the least sacrifice of organs possible, with the greatest safety possible, and that will restore permanently the health of the patient. These I would make the basis of an operative procedure. We never have had the results of vaginal operations for pelvic suppurative disease recorded in this country. When we get that history I believe the number of injuries to the bowel and bladder that have occurred will be astonishing. If you operate suprapubically and injure the bowel and the bladder and do not repair them, the subsequent danger to the patient's life is very great. In operating by the vagina all kinds of surgical sins can be committed and the patient's life may not be paid as a penalty. That appeals strongly to many operators.

An objection made to the suprapubic method is that it leaves a scar. I appeal to the Fellows here who have operated by the abdominal method, and ask if they have ever yet heard even one woman make any complaint of the scar? Personally I cannot recall a single instance. Again, immunity from hernia following vaginal operations is given as an argument in its favor. I think when we come to know the facts regarding vaginal operations we will find that a great many more hernias occur as a result of this method, together with prolapse of the vagina, than by the abdominal route.

The next objection to the abdominal operation is, that by leaving the uterus it is a focus of infection and the patient is not well on account of it; and another argument in favor of removing the uterus, after the tubes and ovaries have been taken out, is that it is no longer of any use. I do not think either one of these arguments will be found tenable.

I believe the number of operations done by the vagina for pelvic suppurative disease is diminishing. Many operators have started with the vaginal method, and before completing the operation have resorted to the suprapubic method, showing conclusively by their own technique that we have a more advantageous field of manipulation from above. Furthermore, advocates of the vaginal method decry drainage. They say it is absolutely not needed and should be abandoned; that it is a disadvantage in that it produces hernia and makes holes in the intestines. They then claim that better drainage is afforded by the vaginal route. There is very little logic in such arguments. It is doubtful if some of the fundamental principles of surgery are ever improved upon; as, for instance, Ambrose Paré's discovery of the ligature. We may change the material, but the method of tying an artery and of rupturing its coats so as to secure permanent closure is something that will probably remain in surgery forever. When we come to remove disintegrating and sloughing tissues, we should take them away thoroughly and completely, separating all adhesions and restoring normal relations. In these cases we apply a great principle of surgery which is as everlasting as the tying of an artery.

There is a field for vaginal hysterectomy in certain puerperal cases. There are cases of puerperal sepsis in which the uterus is riddled with small abscesses, where that organ is undergoing sloughing, and where the patient is reduced to such a degree that she should not be subjected to a very extensive operation. In such cases I can readily see that to quickly clamp everything to save time, to remove the uterus and establish drainage in that way, would be a procedure that has a field of application which is legitimate and valuable. But we have seen during the past two or three years hysterectomy done as a routine procedure. In my opinion it is bad surgery and I do not think it will last.

In regard to the other method of procedure, I do not see any reason to make a change. Nothing is more satisfactory in a case of suppurative salpingitis than to open the abdomen, turn out the masses, tie them off closely, separate all adhesions, clean out all dirt, insert a drainage-tube and put the patient to bed. No recovery is more prompt, no procedure more rational, and no result more permanent and satisfactory.

Dr. JOHN M. DUFF, of Pittsburg: I was one of the last in my region to begin vaginal hysterectomy. I commenced to do it, perhaps, a little against my own judgment, because everybody else was resorting to the operation, and it became a fad. I operated on quite a number of cases by this method and my patients did well; in fact, they did so well that for some time I did not resort to any other method until the meeting of the American Medical Association at Atlanta. After listening to papers and discussions there I went home with my mind made up to do both operations—that is, I would put the patients in bed, side by side, and endeavor to ascertain the results. My operations since that time have not been great enough in number to draw such conclusions as I would like before expressing an opinion.

There is one important factor in connection with this subject which I have not been able to definitely decide for myself, nor has anyone else satisfied me fully—namely, with regard to the propriety of allowing the uterus to remain after removal of the appendages. There are some good reasons why, perhaps, it should remain. On the other hand, if allowed to remain it is a focus for sepsis, and as far as the condition of the woman is concerned, the results, as attested to me by my patients, are that they feel as well as do those who have had their uteri left *in situ*. I have been fully satisfied with one question that was brought up at the Atlanta meeting, which is that patients enjoy sexual intercourse fully as well, and two patients upon whom I have operated since that time, who did not have much sexual appetite prior to operation, have noticed an increase of the sexual passion since then. One factor of which Dr. McMurtry speaks as in favor of the suprapubic operation, is that the surgeon can always see what he is doing, and there is perhaps much less danger of injuring the intestine; but, on the other hand, there is not as much shock to the patient attending the vaginal operation. As a rule, when I operate by the vaginal method my patients rally sooner than they do after the suprapubic operation. They are able to be out of bed sooner than those upon whom I do a suprapubic operation.

I asked one of the internes of the hospital in Pittsburg to send me the temperature records of some cases upon which I had operated, and through a mistake they got mixed, hence I have only three of them in my pocket, and they happen to be the temperature records of the vaginal hysterectomy cases. I am sorry I have not obtained the suprapubic temperature records to compare with them. I will pass these three around. In one case the temperature rose 1° or 2° ; in the others there was comparatively no rise of temperature at all. The patients felt good and wanted to sit up in bed two or three days after operation.

Dr. STONE : Was there a rise of temperature in any of your cases before the operation ?

Dr. DUFF : Yes. Two of them had considerable temperature ; the other did not. One patient had a temperature of 104° when operated upon. On the second day after operation it came down to normal and remained so. We ought to be fair. I am trying to come to just conclusions in regard to the relative merits of the two operations. I remember a case two years ago that was brought into the hospital upon which I was afraid to operate, the patient having a temperature of 105.5° when placed on the operating table. I did a suprapubic operation, and within two or three days the temperature came down and never went up thereafter, the woman making an excellent recovery. Dr. McMurtry has intimated that it does not require as much skill to go in through the vagina as it does to do suprapubic work. It seems to me the factor in deciding the particular method to be pursued is a man's ability. Naturally he will resort to that method by which he can do the best work, and I believe I am among the number that do their best work through the vagina.

THE PRESIDENT (Dr. Joseph Price) : I agree with Dr. McMurtry in regard to the importance of the three papers that have been read, and we cannot devote our time more profitably than to a discussion of the relative merits of the vaginal and abdominal operations. At the meetings of the American Medical Association, where so many different views are presented and where the general practitioner goes to get light, he really becomes confused. Dr. Davis is right in his position, but it is a difficult matter indeed for him to give you his precise position. For instance, he told you that in the desperate, dying class of patient—those that were almost helpless, with pelves full of pus, —we should resort to incision and drainage. While this method may afford temporary relief, a more radical operation will be called for later on. A great many cases where the vagina is incised and drained are placed on record as cures. If you incise a right iliac abscess and a left pus-tube, leaving the right ovarian abscess and right pus-tube, you leave half of the pus behind. You have only half-drained. There will be an old, necrotic sac in the deep pelvis, and you should not leave it. You stick gauze in there and call it a cure. It is not a cure. In from one to fifteen years later the woman has a sinus or sinuses in the sacrum or groin, and then it is a hopeless case. Some of these patients are subsequently treated for sepsis or typhoid fever.

Twenty years ago, in one of the Philadelphia hospitals, the vagina of a woman was incised and drainage established, followed later by a suprapubic section, with the drain pulled through and tied around the pubic arch. One patient's vagina was incised several times by different Philadelphia surgeons; she went to Pottsville, and fifteen years after the incision there were seven sinuses about her sacrum and groin; through a fresh incision I evacuated a gallon of pus and established complete drainage. We must not lose sight of the history of pelvic abscesses by the old treatment of vaginal incision and drainage, and this is only a revival of it that we have had in most of our meetings. We have held fast to most good things in surgery, and if our forefathers had found the puncture and stab methods good, we would not have rejected them. I am sorry that Kelly reports thirty-seven cases of vaginal incision and drainage with one death; and just here I wish to touch upon a vital point: hospital surgeons at the present time are afraid of their colleagues and are also afraid of their board of directors. Their colleagues and the board of directors are now familiar with the methods of operating and the mortality following them. You will find surgeons throughout the country dismissing a class of cases that some of us have to care for. I have passed through one city in this country eight times to do operations rejected there as inoperable or hopeless. I lost three cases out of the eight. A surgeon recently said to his assistant: "We will have to be careful; we have had too many deaths, and the managers are complaining." He closed the incision, turned the patient over, and punched a little hole in her vagina for drainage. If a surgeon is afraid to open the abdomen for the purpose of enucleating huge pus-tubes and ovarian abscesses and repairing lesions of the sigmoid, freeing sixteen inches of ileum and repairing it if necessary, he should adopt the vaginal route, or send all such cases to a man with thirty-three vertebræ.

I look upon vaginal surgery as absolute play. Extirpation of the uterus by the vaginal method is one of the easiest operations in surgery. Enucleation of pus-tubes and ovarian abscesses in cases of long standing is not so easy ; they are not enucleated by the vaginal method. We must consider work done by the vaginal route as incomplete. Here I will refer briefly to Jacobs's cases. In seventy-two cases he did two abdominal sections, in one of which he used the Murphy button, doing good surgery, and saving his patient. In the other he removed the kidney for ureteral fistula. Secondary operations have been quite numerous of late as the result of incomplete vaginal work. The vaginal operation is not sufficiently old to give us a group of cases that will turn up in the hands of others for completion. I must insist that vaginal operations are imperfect.

Someone has alluded to pelvic abscess. I have done nearly 4,000 sections, and I have not seen pelvic abscess independent of tubal and ovarian disease, except it was traumatic. A crochet-needle inserted through the vaginal vault may cause a broad-ligament abscess. We may have abscess in the cellular tissue due to traumatism, but I have never found one due to inflammatory mischief.

Reference was made to Dr. Noble's cases as errors in diagnosis. Three or four of them were cases of appendicitis, and two or three were pus cases with secondary operations for removal of the pus-tubes, and one a suppurating extrauterine pregnancy with the fetal bones sticking through the vaginal vault. Dr. Noble's cases were (nine) errors in diagnosis. Vaginal incision and drainage is an old operation ; there is nothing new about it. Extirpation of the uterus, as practised, is a simple procedure, and in America I do not think our results are any worse than they are abroad. I condemn the vaginal route, not because my mortality influences me in the least, for I have a series of vaginal hysterectomies of fifty-three cases with one death, and in this series I removed everything. It is a safe operation, but an incomplete one. The surgeon remains in perfect darkness as to what exists above.

Dr. McMurtry has alluded to appendicitis complicating tubal and ovarian disease. In every hundred abdominal sections for suppurating tubes and ovaries, we remove the appendix in 6 per cent. of the cases. I removed it day before yesterday in a case, and also twice last week. I removed it along with the suppurating tubes and ovaries. For instance, in the last twelve days I have stitched the bowels seven times in suppurative forms of disease. Can you imagine anyone adopting the vaginal route with such a mental picture before him ? Only ten days ago I operated on a case where, if I had selected the vaginal route, I should have overlooked two lesions. A few days ago a physician in operating on a case of appendicitis found an adherent infected omentum, which was not removed. He lost his patient and then regretted that the infected portion of omentum had not been removed. Abscesses and the like are commonly overlooked where the vaginal route is resorted to. Four days ago, while examining a patient, I recognised a twisted pedicle. The common history of twisted pedicle is pain, rapid increase in the size of the tumor ; and I said to the doctor by the side of me, " we have a twisted pedicle to deal with." I opened the abdomen, found an ovarian cyst in the bottom of the pelvis, found the omentum adherent to the tumor, and after reconnoitering I thought we had the left kidney or spleen, but, after freeing the omentum, it turned out to be a tumor as large as a child's head growing from the small bowel. I did a resection of the bowel, then removed the ovarian cyst. Suppose I had done the vaginal operation, I should have removed the small tumor and permitted the malignant tumor of the bowel to remain.

" Septic uterus, rotten uterus "—these are peculiar terms. If there is a single woman in this city with a " septic " or " rotten " uterus, she will be in glory in a few hours. Sepsis is the same the world over. It kills quickly. " An infected, rotten uterus." You commonly hear men using these expressions while criticising some one else's work. It is very curious, but a few years ago the same class of men would remove a tube and ovary, and the woman afterward would bear children. Now they say the uterus is "septic" and it must come out. A few years since, operators recorded a series of fifty and 100 cases with the most happy results. But now the results are all bad. The man who does a complete operation may, perhaps, have an additional mortality of 3 to 5 per cent.

As to insanity, we have not in this country now what may be termed a surgical asylum for the insane. It is folly for us to talk about this matter with a lot of politicians who are at the head of our institutions. The man who will open a private asylum of this character will do the best work that can be done for the women of our country.

I have repeatedly found mesenteric abscesses and growths above the pelvis in my surgical work, independent of pelvic disease coëxisting. In pelvic surgery the removal of pus-tubes and ovarian abscesses is the simple part of the work. The leaving of a healthy and normal condition above, after removing the pathological conditions and lesions present, is really the important part of our work, and it is absolutely impossible to do complete surgery by the lower route.

Dr. J. Henry Carstens, of Detroit: Dr. Dunning reported a number of cases that he had operated on, among which were three cases where the uterus was diseased, and which he thinks he will have to remove after having performed pus-tube operations. I am somewhat in the same position. I have operated ten times to remove the uterus where either myself or somebody else had previously operated for diseased tubes. The uterus was diseased. There was a degenerated condition of the mucous membrane. The hemorrhage and nervous symptoms continued, and two of them were the wives of physicians. Formerly some physicians did not believe in the removal of a pus-tube, but now they say that if the uterus is diseased also, you had better remove it at the primary operation, if you think it can be done all at once. So, gradually, we have been drifting around from one side to the other.

Dr. Dunning says that some of us have been bitterly attacked. I think no one has been more bitterly attacked than Dr. Price, and each one of us knows what it is to be attacked. Dr. Price may be considered as a pioneer in this kind of work. No one, however, has a right to condemn the vaginal method of operating and say that it is incomplete. He cannot say it is incomplete and that the ultimate results cannot be good until he has tried it. All innovators have been abused or ridiculed and hounded in every possible manner. Such men as Hegar, Tait and Battey have been vilified in every possible way. We have all been called reckless and considered as men without conscience. Péan, Segond and others have been subjected to the same abuse. This is not fair. These men are not all liars, and if they positively assert that they have had so and so many hundred cases which were successful, their assertions should count for something and their suggestions should be tried. After a fair trial every method will find its level.

I believe that vaginal hysterectomy for pus-tubes will show a smaller mortality and give better ultimate results than abdominal section. That is my present belief. I think cases should be selected, however. I believe in conservatism. In a young woman, I believe in abdominal section and an effort to save, if only one-half a tube and one-half of an ovary; while in women near the menopause, vaginal hysterectomy should be the rule. I wish to be distinctly understood that I am decidedly in favor of vaginal hysterectomy in certain cases, and I do not believe that all cases should be subject to abdominal section; but that one class of cases should be subject to one operation and another to the other. Nothing should be condemned upon theoretical grounds. No one has a right to assert things about which he knows nothing. First try them honestly and faithfully. The surgeon should understand the technique of both operations.

Dr. Albert Goldspohn, of Chicago: It has been my experience that vaginal hysterectomy is an easy operation. I feel very kindly toward it; but because it is easy is not a sufficient reason for sacrificing the uterus. There is a vast difference whether a woman knows that she loses the uterus for carcinoma or for some other disease which she knows will not kill. Cancer is a terror to women, and when a woman knows that she has had a carcinomatous uterus she does not doubt that she has been blessed in its removal. But when we remove this organ for other causes, such as an inflammatory disorder for instance, the patients often dote over their imaginary loss, and what we call sentiment, with them is a mental pain that borders on mental aberration, and sometimes leads to it.

During the last winter a deep impression was made upon my mind by being terrified for several months at the prospect of two of these cases on the verge of going into an insane asylum. It took great care on the part of myself and a neurologist to keep them out.

The question regarding the abdominal or vaginal method of operating cannot be definitely settled in favor of either procedure; but as surgeons we must occupy a middle ground and use judgment. There are some cases in which the uterus should be removed, and if operated upon at all it should be attacked from below. On the other hand, if we have to deal with large pus-tubes with the intestines adherent to them, it is not easy to attack such a case from below. I have tried it, and know what it is. The total absence of sight, in such cases, the relative inaccessibility and the exceedingly strained palpitation endanger the patient more from the consequent bad or imperfect surgery than does the shock from a careful but thorough and complete ventral operation.

Dr. George H. Rohé, of Sykesville: I did not intend to take part in this discussion this afternoon, but several statements made in the papers ought not to go out without an attempt being made to call them somewhere near the middle line. In the first place, I would like to speak of the removal of the uterus from below. A good many years ago Dr. N. R. Smith invented an instrument by which lithotomy could be easily done, and some of the surgeons in Baltimore objected to it because "any —— fool could do lithotomy now." The trend of the argument in this discussion has been that almost any kind of a fool can remove the uterus through the vagina. To me it is a good deal easier than to remove it from above, and that is the reason I prefer to do it in that way. I believe it is the method to pursue, and that it is just as good for the patient. In the few instances in which I have operated in this way I think it has been better for the patient than if I had operated by the abdominal route.

The statement has been made in the papers read here that women who have the uterus and appendages removed were in great danger of becoming insane; that the nervous phenomena were very much more severe than when the ovaries and tubes were alone removed. I would like to know where the facts are to be found upon which this statement is based. My own observation does not support this view.

Dr. James F. Baldwin, of Columbus: I think the gist of the papers has been lost sight of—namely, whether in removing the appendages we shall resort to the abdominal or to the vaginal route. A discussion of this subject is threshed straw; it was considered by us a year ago at Chicago; it was discussed at the Atlanta meeting of the American Medical Association, and is, indeed, a perennial theme. The advantages of both methods have been considered. One point to be discussed is whether we shall remove the infected uterus when we take out the appendages, or whether we shall not. Dr. Dunning tells us that he has operated on 116 cases, has removed the appendages and left the uterus.

Twelve he reports as not well; three were distinct failures and will have to be operated on later for removal of the uterus. A friend of mine has corresponded with a large number of operators throughout the world, and has accumulated several thousand cases of operations for removal of the appendages. In 5 per cent., as an average, there has been failure to effect a cure; 95 per cent. are well with their uteri. I have no doubt that the complaints of the 5 per cent. of failures have given the operators infinitely more annoyance and anxiety than the praises of the 95 per cent. of the cured have given satisfaction.

For several years I have been in the habit, where the uterus was found distinctly diseased and enlarged, or where there was a history of long-continued trouble, of taking it out. I have never regretted it. I have never known a woman go insane or complain of loss of sexual appetite. I have found the sexual appetite just as good as before operation, and in a number of cases a good deal better; not, I mean, better than it was immediately before the operation, but better than when the parts were normal. To demonstrate the condition of the uterus in these cases I frequently take that organ, just as I have removed it, and give it a thorough curetting before the class. I then split it in two and show how little effect the curetting has had. The tension in these cases is so great that the uterus, when thus split, opens very much like a grain of popped corn. I not infrequently refer to them as cases of popcorn uterus, and I do not believe that any amount of curetting, or carbolic acid, or iodised phenol, will restore such uteri to normal conditions. Hilton, in his work on Rest and Pain, an exceedingly valuable book, alludes indirectly to this class of cases when he speaks of tension as giving rise to pain, and I think that this tension is largely the cause of a good deal of the pain and other nervous symptoms that our patients complain of.

As to the vaginal route, I have made a score or more of vaginal hysterectomies. It is a very easy operation. The first one I did was done in seventeen minutes. I was astonished at its ease. But it does not seem to me surgical. When I have made an abdominal hysterectomy, removed all I think there is to be removed, brought the peritoneum nicely together, tightened up the broad ligaments, so as to support the stump of the cervix, brought in the round ligaments also, so that the vagina is held up nicely, and have then closed the abdomen, I feel that I have performed a surgical achievement. When I have operated through the vagina I do not know exactly in what shape things are above. I am trusting largely to Providence. I do not feel that I have done a good surgical operation. Nevertheless, I think there is a fairly broad field for the vaginal operation. I think if I could go over again some of the cases I have operated on through the abdomen, I should go through the vagina for the temporary purpose of improving the condition of the patient preparatory to doing a more radical operation, because I like finally to feel that the patient has been surgically well operated on.

I think the French surgeons have been carrying to an extreme the matter of vaginal hysterectomy for inflammatory conditions.

It may be that we have been doing too much through the abdomen for these same conditions, and that somewhere between lies a golden mean. I am planning now to visit Paris this fall and see these men work, and then draw my own conclusions as to the character of the work itself and the class of cases which would be best subjected to the vaginal operation.

Dr. Walter P. Manton, of Detroit: Between 3,000 and 4,000 insane people have come under my observation, and I do not recall a single instance in which the insanity could be traced to the removal of the uterus or the appendages. In my own experience I have found during the last thirteen years but two instances in which I was sorry that I did not remove the uterus at the time of the primary operation. One of these women had a specific uterus, which has given a great deal of trouble since the operation. The other woman became infected subsequently by her husband, and I think all that trouble would have been obviated if I had removed the uterus. These are the only two cases that I can recall in which the uterus should have been removed.

Dr. J. Wesley Bovee, of Washington (by invitation): I placed myself on record at the Atlanta meeting of the American Medical Association as regards the relation of the two operations, and I simply desire to say that I have not modified my opinion since. We have forgotten a few points in the consideration of this subject which have some bearing upon it. There is often infection of the ligature, which causes trouble. There are frequently injuries to the uterus, or little points possibly that have not drained well, and this gives trouble. It is not the uterus alone which gives trouble, and in some cases after you have removed the uterus nicely you will not have cured your patients entirely. So far as drainage goes, I have not drained through the abdominal opening for a long time, but entirely through the vagina in the manner described.

The President: Imperfect work and imperfect materials have been used as arguments against legitimate procedures. No one should criticise vaginal operations without a working knowledge of both the abdominal and vaginal methods. He ought not to, at least. An argument should never be made against any operation until it has been thoroughly tested. We know there are a number of men in America who are condemning operations, and yet are wholly ignorant of them. Jacobs and Segond are simply masters in their vaginal operations. No one can read Jacobs or hear Segond talk without recognising at once that they are both masters and fully understand their work. But it goes without saying that their work is incomplete. If you can dismiss everything above the uterus, if it is simply a matter of removal of tubes and ovaries and not of correcting the overlying complications, the vaginal route is the route to pursue. If I could dismiss everything above, I would never open the abdomen again. For the removal of the uterus alone the vaginal route is important. My little girl asked to go with me one day to the city, and I said "No, I am too busy." She then asked me if I was going to operate. I replied, "Yes." She is only eight years old. I said to her, "Do you understand what that means?" She replied, "Yes." "What is it?" She said, "Fixing people's insides right." As long as I have to fix people's insides right I shall do the suprapubic operation, and not neglect my duty to my patient by an incomplete procedure or shirk my duty.

Dr. Rufus B. Hall, of Cincinnati: It was not my intention to say anything on this subject, but one or two statements that have been made prompt me to say a few words. Like the other members of the association I believe there is a field for the vaginal operation. We all know what a relief it is to our patients and a gratification to ourselves in vaginal extirpation of the uterus in certain cases of cancer of this organ. After the uterus is taken out they remain well for an indefinite period, and we are much gratified at the result. In the majority of cases it is a perfectly easy operation. We go about it and do it without much trouble. Occasionally we have to separate an adherent ovary that is high up and very difficult, but we peel it out and rid the woman of her trouble. When it comes to removal of the uterus where there is bilateral disease of the appendages, as a routine practice, as some operators claim, I do not think it is the correct method. It is known to be true that a large per cent. of cases of bilateral disease of the appendages get well after having been operated upon by the abdominal route, and remain well, in which the uterus was not removed. I should say the percentage would be somewhere about 95. It is a simpler and less mutilating operation than taking out the uterus, which must be done in the other method for this reason.

I much prefer to do a hundred operations for bilateral disease of the appendages and leave the uterus in every case, and later remove the uterus where it is indicated in the 5 or 6 per cent., if I were compelled to do one or the other. I believe in some instances that it would not be a bad idea if we could decide just when to take out the uterus with the appendages ; but when I do that, I do not do it by the vaginal route, as a rule. I can do it much more easily and satisfactorily to myself by the abdominal route, and then I know I have done a complete surgical operation when I am through. When we resort to the vaginal route with the clamp, and pack the opening with gauze, we cannot see what injury we may have done to adherent coils of intestine or omentum. An adherent coil of bowel to the gauze that is left impacted must necessarily follow in some cases, and is a source of danger to the patient. There are many points I would like to speak of, but as my time is limited I will not do so. The main point with me is that I cannot repair intestinal injury through an opening where I cannot see what I am doing, as I can when operating by the abdominal route. We must necessarily injure the bowel in a given number of pus cases. Our President said that he did so twice last week ; and I did it once, also twice the week before in similar cases. If I had done a vaginal operation I do not think I would have been quite so successful.

Dr. Davis (closing): Many of the points have been covered in the discussion, and it is not necessary for me to dwell on those phases of the subject. The choice of operation will depend largely upon the operator, and it is well, perhaps, that some surgeons are extremists in one method of operating, and others in another. By such extremes we often reach the highest order of work, notwithstanding those who confine themselves to one particular method generally see nothing good in any other method. It would seem that the whole question should hinge upon the disposition of the uterus. What shall be done with it? We understand why the French surgeons resort to hysterectomy. They have operated by the vaginal route more frequently, and have become accustomed to it. The removal of the uterus is the preliminary step to all their pelvic operations. It must be removed to get at the organs. But it is hard to understand how Americans, who are removing the uterus from above, can reconcile this procedure, for it does not facilitate their work in the least. The French surgeon does it because it enables him the better to do intrapelvic operations ; while the American does it—I won't say to increase his number of hysterectomies, because that would not be fair ; yet it affords a splendid opportunity for one who has become tired of simply removing the tubes and ovaries to make a large list of abdominal hysterectomies. There can be no advantage in it. These very men have published statistics in which they claim to cure a large percentage of their cases by the removal of the tubes and ovaries before they adopted this method.

The men who have cut open tubes and injected bichloride of mercury into them, and claimed to have cured so many cases by the curette, are the men who are now advocating this method most enthusiastically. What we want to accomplish first is the conservation of life, and the old practice of incising pelvic abscesses, to bridge the patient over, is a good one. We have all resorted to it. There is not a member here who would not indorse that procedure for a certain form of pelvic abscess, but where the pus is in the tubes or ovaries there is a division of opinion. It is not correct to say that in every case in which we incise a pelvic abscess we should expect to have a reaccumulation of pus; for many are cured. After these abscesses have been drained frequently a mass will be left behind. It is tubal, and if you incise it and pack gauze in the cavity you will destroy it. When we incise the tube we do it for what we would do for an abscess, and the pyogenic membrane of the tube is replaced by granulation tissue, and, finally, the tube almost disappears. My own work warrants me in continuing to incise tubes and ovaries, and I am more and more encouraged to do it on account of the good results of others. Our first duty is to conserve life, and the second is to conserve important organs. It is a beautiful thing to go in and enucleate a pus-tube, but our patients are demanding of us that we save important organs, and where a number of good men record many successful cases by the simple operation of vaginal section in connection with curettement of the uterus, we have no right to do a more formidable operation till we have given our patients the benefit of this preliminary work.

As to drainage, I think this question ought to be settled. It makes no difference whether gauze drains pus or not. If the gauze will drain serum and protect the abdomen for forty-eight hours, you do not care then whether it drains or not. You gradually remove the gauze from the external opening to keep it patent, and the fluid will come away. The abdominal cavity will take care of itself after forty-eight hours. If you remove the gauze sooner than four or five days you are apt to break up adhesions and induce a worse condition than existed before the operation.

As to diminution of sexual desire, I believe that the consensus of opinion is that after a time there is less sexual desire where the tubes and ovaries have been removed, yet we have a great deal of difficulty in ascertaining from patients the facts about this matter, the sexual desire having been destroyed often by the disease for which the operation was performed.

Dr. McMurtry: Before Dr. Davis sits down, I want to ask him to make clear one point, as we have so much respect for his opinion in these matters. I would like to have him explain whether or not, in the class of cases in which he has hitherto done abdominal section for suppurative pelvic disease in women, he has changed his practice, and whether he now substitutes drainage for the operation of abdominal section; whether he is applying the principle that he has hitherto observed, or whether he has adopted a new procedure in his practice?

Dr. Davis: In reply to Dr. McMurtry, I will say that I have practised what he and all the rest practise—namely, to incise and drain pelvic abscesses. I realise that they have their origin in the tube, and after incising such abscesses I expected afterward to do a radical operation when the patient was in a better condition. Now, I simply incise these abscesses, and examine my patients weeks afterward, and if I find a considerable mass connected with the ovary or tube, instead of doing an abdominal section I resort to vaginal incision. I do not curette the uterus at the first operation, believing that the patient should not be taxed that much. I reserve curettement for the second operation, when I incise the mass containing the tube or ovary, and pack it with gauze. After a short time the tube will be obliterated. In many of these cases a radical operation is saved.

Another point: the adhesions of which Dr. Price spoke do not remain, particularly in the acute puerperal cases. They are like appendiceal adhesions. You drain an abscess of the appendix, the appendix is drained, and you do not have to do another operation. Should you open the abdomen a few months afterward, you would find scarcely any adhesions. In the puerperal cases of pelvic abscess, where we have to incise large collections of pus, the adhesions are very extensive, and it is not wise to break them up at the first operation. In other words, it is nature's method of protection, and we should let nature take care of them. In the second operation you will have to break up some very firm, well-organised adhesions, but this is a small matter when you have only the tubes and ovaries to deal with.

The President: Dr. Davis refers to a class of cases in which he resorts to vaginal incision and drainage. If the patients are not relieved by this method of treatment they generally go to some other practitioner. I may say that 90 per cent. of the cases where this treatment alone is carried out are not well, and they go to other surgeons. For instance, during the last ten days ten patients have come to my hospital for a second or third operation. A good number of them have been timid, incomplete procedures.

Dr. Dunning (closing the discussion on his part): I have very little to say in closing the discussion. There seems to be a misapprehension regarding some statements I made. I distinctly stated that it was entirely justifiable to remove a malignant or neoplastic uterus. This answers the question of Dr. Carstens. It is a life-saving operation.

Regarding the question that was asked by Dr. Rohé, I stated that but one instance out of eighty-four cases of hysterectomy which I have done showed any signs of insanity afterward, and she had evidences of it before operation.

With reference to temporising in cases of acute pelvic abscesses, it has been my practice for twenty years to do this. I have incised at least twenty abscesses during the last year. Of this number I have had to do a secondary operation in three instances. I have watched some of my patients for fifteen years after incision of a pelvic abscess, and in many there has been no recurrence of the trouble. These patients were my friends. It is perfect folly to say they did not get well. I have delivered some of them of babies since. Neither Dr. Price, Mr. Tait, nor anyone else can convince me that they do not recover. Of course, a certain percentage of them will have to be operated on again. This is as near the truth as I can get. No argument can upset my statements in this regard. There is no question but that we ought to do thorough work.

Finally, I want to say that I have admired our President ever since I have known him. He has been an inspiration to everyone who has observed his successful work. His work, too, has been instructive to me. I have watched every report he has made, and have been guided largely by his work, but I differ from him on this point. His conviction that the idea he advances upon this subject is the ultimate truth is no stronger or more deeply rooted than is mine, that to pursue the course he advocates would with the average man lead to the sacrifice of many lives, which, if the lesser radical method were adopted, would be tided over and carried beyond the danger-point, and would ultimately be restored to health.

Dr. Fish (closing the discussion) : Some two or three weeks ago I called the attention of our local society to the fact that uteri were seemingly being removed in a wholesale way. In my region of the country it has often been remarked that we take out the uterus first and make the diagnosis afterward. If this association advocates the removal of the uterus every time we take out the tubes and ovaries, it is going to lead to wholesale hysterectomy all over the civilised world. When such a man as Schauta, of Vienna, says that whenever we find a unilateral pus-tube due to gonorrhea, we must remove the other tube and uterus also, it seems to me he is going too far.

Again, it has been remarked that when the uterus is left, after removal of the tubes and ovaries, we frequently have hemorrhages or purulent discharges which do not yield to curettage or cauterisation, and that at last the uterus must be removed. While this may be largely true, if only 50 per cent. of the patients recover, is it not better for us to leave the uterus ?

With reference to insanity, I do not claim that I have ever seen a case of insanity follow hysterectomy, but I have had cases of mental "blues" or mental depression in women who seemed to think of nothing else except the fact that their uteri were gone, and I do not know what it will lead to later on.

I have had patients with carcinoma of the uterus who had the mental blues before their uteri were taken out, and who felt mentally improved after they were removed. I have done vaginal hysterectomy sufficiently often to satisfy myself that it is a simple procedure by the clamp, and I shall continue to use the clamp in cases of carcinoma of the cervix until I find I have a large number of cases of prolapse of the vagina as a consequence, when I will probably stop. For high pus-tubes and other pathological conditions of the pelvic organs I shall continue to operate from above.

---

Haig now claims that uric acid is derived largely from animal foods, hence the diseases resulting from an excess of the acid in the system can be controlled and prevented by proper regulation of the diet.—*Denver Medical Times.*

